# Predictors of Vaccine Hesitancy: Implications for COVID-19 Public Health Messaging

**DOI:** 10.3390/ijerph18158054

**Published:** 2021-07-29

**Authors:** Amanda Hudson, William J. Montelpare

**Affiliations:** 1 Department of Mental Health and Addiction, Health PEI, Charlottetown, PE C1C 1M3, Canada; 2 Department of Health Management, University of Prince Edward Island, Charlottetown, PE C1A 4P3, Canada; 3 Department of Applied Human Sciences, University of Prince Edward Island, Charlottetown, PE C1A 4P3, Canada; wmontelpare@upei.ca

**Keywords:** vaccine hesitancy, individual differences, public health, COVID-19

## Abstract

Objectives: Successful immunization programs require strategic communication to increase confidence among individuals who are vaccine-hesitant. This paper reviews research on determinants of vaccine hesitancy with the objective of informing public health responses to COVID-19. Method: A literature review was conducted using a broad search strategy. Articles were included if they were published in English and relevant to the topic of demographic and individual factors associated with vaccine hesitancy. Results and Discussion: Demographic determinants of vaccine hesitancy that emerged in the literature review were age, income, educational attainment, health literacy, rurality, and parental status. Individual difference factors included mistrust in authority, disgust sensitivity, and risk aversion. Conclusion: Meeting target immunization rates will require robust public health campaigns that speak to individuals who are vaccine-hesitant in their attitudes and behaviours. Based on the assortment of demographic and individual difference factors that contribute to vaccine hesitancy, public health communications must pursue a range of strategies to increase public confidence in available COVID-19 vaccines.

## 1. Introduction

Since the first cases of coronavirus, SARS-CoV-2, emerged over a year ago, the virus has had devastating global effects. As of 28 March 2021, cumulative worldwide cases of coronavirus 2019 disease (COVID-19) reached 127 million, and the number of deaths totaled 2.78 million [1]. As of March 28 2021, the total number of cases and deaths in Canada had reached 961,000 and 22,852, respectively [2]. Prior to vaccination rollout, containment efforts relied primarily on public health measures, such as social distancing, self-isolating, travel restrictions, hand hygiene, mandatory or recommended mask-wearing in public, widespread testing, and, when necessary, lockdown procedures [3,4].

International research efforts advanced at an unprecedented speed in the pursuit of a safe and effective vaccine. With Health Canada approving the Pfizer-BioNTech COVID-19 vaccine 9 December 2020, the Moderna COVID-19 vaccine 23 December 2020, and the AstraZeneca and Janssen vaccines 26 February and 5 March 2021, the next step in mitigating the COVID-19 pandemic is the widespread administration of approved vaccines. For priority groups in Canada, COVID-19 vaccination began in late 2020 to early 2021 with broader availability to the general public to follow throughout 2021 [5]. Careful steps must be taken to increase readiness among the population to maximize uptake of the vaccines.

The success of a vaccine depends not only on scientific and clinical readiness (i.e., having an adequate supply of a rigorously tested vaccine) but also on public readiness (i.e., intent among a large proportion of the population to be vaccinated, conferring herd immunity) [6]. Traditionally, there have been two main threats to successful immunization programs: failure to vaccinate, which is closely related to attitudes and behaviours of the population, and vaccination failure, which reflects the (in)ability of the vaccine to build appropriate immunity to the virus [7]. Current vaccine efficacy rates are high, 95% and 94% after both doses of the Pfizer-BioNTech and Moderna vaccines, respectively [8,9]. However, widespread immunization will require more than accessible and effective vaccines; it will require an understanding of factors that contribute to vaccine hesitancy and an application of this knowledge to inform targeted communication efforts [10]. In effect, assessing factors related to the public endorsement of a COVID-19 vaccine is central to informing a meaningful public health response.

The scale of vaccine rollout will depend on the decisions of individuals who range from vaccine confident to vaccine hesitant. This challenge requires that public health officials move away from a one size fits all strategy to enhance vaccine confidence among individuals that may be vaccine hesitant. In other fields of public health, including substance use prevention, selective programs for at-risk populations have had success in terms of intended outcomes and cost-effectiveness, whereas universal programs fall short [11,12]. Vaccine campaigns should follow suit and target individuals with known risk factors for vaccine hesitancy. For context, surveys by the Angus Reid Institute (December 2020) found that approximately 45% of Canadians indicated they would not get vaccinated immediately or at all upon the availability of a vaccine. Similar rates of COVID-19 vaccine hesitancy have been reported in the U.S [13]. The most recent Health Canada data indicate that 13% of the population are fully vaccinated, and 51% are partially vaccinated. Yet, some pockets of the population remain hesitant. Vaccine hesitancy is distinct from anti-vaccination ideology in that it falls along a continuum, is dynamic and plastic, and is less polarizing [14]. Thus, vaccine hesitancy represents an important target for public health campaigns, as it is malleable as opposed to fixed and holds the potential to bolster public vaccination uptake by focusing on those in need of reassurance.

The World Health Organization has identified vaccine hesitancy as one of the 10 greatest threats to public health (WHO, 2019), highlighting the need to study, understand, and target this construct [15]. Past research on vaccine confidence has indicated that vaccine-related attitudes vary significantly across demographics and are associated with specific individual difference profiles. Global health movements have called for the equitable distribution of COVID-19 vaccines [16]. Part of equitable vaccine distribution is strategic communication to increase confidence among priority populations. The objective of this review is to summarize current research on determinants of vaccine hesitancy and to discuss these findings in terms of implications for COVID-19 vaccine communications. Strategies for developing targeted public health campaigns are presented, and knowledge gaps are identified.

## 2. Method

**Figure 1:**
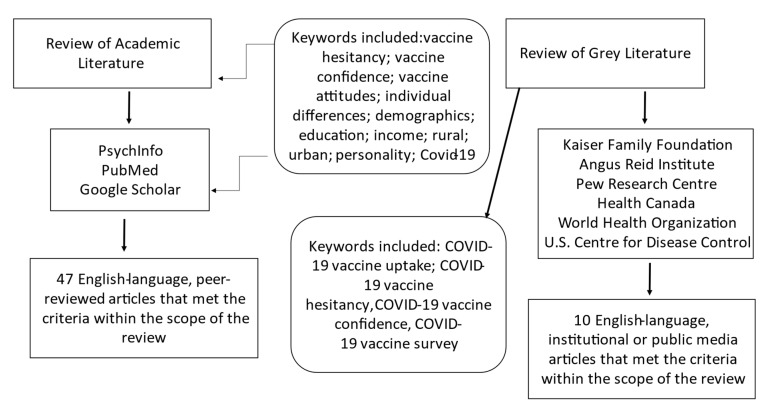
Search strategy for selecting articles from the academic and grey literature.

A literature review was conducted using a broad search strategy with the keywords: vaccine hesitancy; vaccine confidence; vaccine attitudes; individual differences; demographics; education; income; rural; urban; personality; COVID-19 and using multiple databases (i.e., PsycInfo, PubMed, Google Scholar). Articles were included if they were published in English, appeared in peer-reviewed journals, and were relevant to the topic of demographic and individual factors associated with vaccine hesitancy. Survey data released by national organizations or published in reputable media outlets were included to supplement the scientific literature. The grey literature search included specific terminology to retrieve the most recent and emerging data: COVID-19 vaccine uptake, COVID-19 vaccine hesitancy, COVID-19 vaccine confidence, and COVID-19 vaccine survey. Articles contained within the review spanned from 2006 to 2021. To be as comprehensive as possible, we included papers that pertained to the interrelated constructs of vaccination uptake, attitudes, and hesitancy/confidence. Please see Figure 1 for a flowchart summarizing the search strategy. Demographic determinants of vaccine hesitancy included age, income, education and health literacy, rurality, and parental status. Individual difference variables included mistrust in authority, disgust sensitivity, and risk aversion. Findings are summarized in Table 1.

## 3. Results and Discussion

### 3.1. Age

Research has indicated that vaccines are understood and approached differently among different age groups. For instance, correlates of vaccine uptake for the influenza vaccine vary across age groups, with self-reported susceptibility to disease uniquely predicting vaccination in older adults and perceived effectiveness predicting vaccination in younger adults [17]. Along with endorsing different reasons for vaccinating, age cohorts exhibit different rates of vaccine confidence and uptake. Research by Luyten and colleagues found that adults aged 50 to 59 had more confidence in vaccines than those aged 20 to 29 [18]. Age has also emerged as an important determinant of vaccination attitudes and behaviours among cohorts of parents with young children. In a sample of Malaysian parents with children under 7, younger parental age correlated with vaccine hesitancy, as determined by scores on an adapted Parent Attitudes about Childhood Vaccines questionnaire [19,20]. Related to age is the finding that increased social media use consistently predicts more negative vaccine beliefs [21,22]. Given that centennials and millennials are the primary consumers of social media platforms (i.e., Facebook and Instagram), these generations may be disproportionately affected by negative vaccine content [23].

Observed age group differences in vaccine hesitancy have connotations for developing effective vaccination campaigns. For instance, findings of increased hesitancy among young adults indicate a need for communications targeted at those cohorts. Knowing that this demographic group is most affected by social media and that social media platforms are regular outlets for anti-vaccination sentiments and fear-mongering, government and health authorities should bring balance by posting evidence-based content that addresses unfounded fears [21,22]. Internet technology and mass media are conducive to the spread of misinformation but, with strategic efforts, they can also communicate accurate information to targeted groups in ways that effectively counter propaganda.

### 3.2. Socioeconomic Status

A number of studies have investigated income as a determinant of vaccine confidence, although the direction of association has diverged across studies. American families who refused vaccinations for their children, based on medical records, were more likely to reside in communities with higher household incomes than families who did not refuse vaccination [24]. Likewise, a U.S. study with Utah parents found that the most vaccine-hesitant parents were middle-class and held some college education or a college degree [25]. Therefore, affluent households and communities are not immune to vaccine hesitancy, quite the opposite based on the aforementioned findings. Conversely, others have found that higher income promotes vaccination based on the health records of children in rural West Africa [26]. Relatedly, Wu et al. found that low income was associated with less trustful attitudes toward vaccination in post-partum mothers in the U.S [27].

Explanations for vaccine hesitancy among lower socioeconomic (SES) households have emphasized mistrust in the health system, whereas factors underlying relations between high SES and vaccine tendencies are less clear [27]. Higher SES may be associated with improved knowledge of vaccines, based on the assumption that SES is associated with education attainment and, in turn, a general understanding of the relative safety and effectiveness of vaccines. The finding that higher SES is sometimes associated with vaccine refusal is less straightforward but may centre on clean living values, which emphasize purity and natural immunity and tend to be more pronounced among higher-income regions [28]. Moreover, middle- to high-income areas may have more ready access to information through the Internet and social media, which could lead to more exposure to anti-vaccine content [29]. These findings suggest that vaccination campaigns should not be reduced to targeting communities based on SES, as vaccine hesitancy is an issue across social classes. Future research is required to explain why income relates differently to vaccination behaviours and attitudes in different samples.

### 3.3. Education and Health Literacy

With regard to education, results from the Canadian Childhood Immunization Coverage Survey showed an inverse association between educational attainment and concerns over vaccine safety [30]. Comparable findings arose from a study with Greek parents where paternal education of high school or higher uniquely predicted age-appropriate immunization [31]. Lower education levels may deter individuals from vaccinating due to gaps in knowledge about the effectiveness and safety of vaccines or due to inflexible anti-vaccine attitudes [32]. Indeed, health knowledge, in general, is associated with more favourable attitudes toward vaccination [33]. Yet mixed results have been reported, with some studies in the U.S. identifying higher educational attainment as a deterrent to immunization [24]. However, in this study, education was assigned to each case based on their community rather than their household, which introduces imprecision and may confound interpretations. Still, others have found education to be unrelated to vaccine hesitancy in large-scale studies in low- to middle-income countries [34]. Explanations for the mixed findings regarding education and vaccination may resemble the above-noted reasons for disparate associations between income and vaccination.

Educational campaigns may be effective means for addressing knowledge gaps and correcting misinformation surrounding COVID-19 vaccines. Such campaigns might describe a vaccine as a formulation that includes a synthetic form, a dead form, or a weakened form of the disease-causing agent [35]. This active agent is referred to as the antigen and, because it contains the material that looks like the disease, it stimulates our immune system to make new molecules, the antibodies that fight the antigen. Through inoculation with a vaccine, we establish disease-specific immunity. Vaccines enable us to produce antibodies that will circulate in our blood to knock out disease-specific antigens [36].

The Centre for Disease Control (CDC, 2016) emphasizes the importance of using everyday terminology in public health campaigns. As such, a palatable analogy might be to describe vaccines as the personal trainers for our immune systems who enable us to distinguish very specific structures of bacteria or viruses and prepare our bodies to engage in a targeted response. Public education should underline that vaccines are essential to maintaining positive states of health because they assist the immune system to recognize and differentiate which of the 100 trillion life forms, such as bacteria and viruses, that exist in and on our bodies are harmless and which can cause illness and even death [37].

As noted above, education and health education, specifically, are significantly related to vaccine intentions and uptake [33]. When communities are immunized, they reduce the risk of pathogen (virus, bacteria) spread and, in many situations, increase the opportunity to eradicate the disease [38]. Indeed, life before the development of vaccines meant that children and young adults were at constant risk of death and infirmity from diseases such as measles, polio, tuberculosis, diphtheria, and rubella. Today, these diseases are considered vaccine-preventable and, in developed countries, are virtually non-existent when the population participates in public health immunization programs [35]. By educating the public about the history of transmissible disease and the role of vaccination programs in controlling outbreaks, we create pertinent conversations that are relevant in combatting the COVID-19 pandemic.

One of the greatest confounders to the population’s understanding of the urgency to act to control the novel coronavirus has been health literacy or lack thereof [39]. When we consider literacy, and more specifically, health literacy, we are not simply thinking in a dichotomous way about whether or not an individual can read and write. Rather, to be health literate refers to an individual’s ability to understand basic health information and to process that information in a way the enables them to make appropriate decisions related to achieving positive health outcomes [40]. We are now, more than ever, aware of the importance of infectious disease as a major cause of death and disruption in societies. Yet, the pandemic has done more than merely expose the risk of death due to infection. The pandemic has exposed the problems related to the health of and within our society.

While informational campaigns may be effective for individuals whose vaccine hesitancy stems from knowledge gaps, a significant subpopulation may have an adequate understanding of vaccines but may be hesitant for other reasons. Indeed, Volkman et al. found that, for college students, vaccine outreach efforts should focus on students’ perceived risks and fears rather than their level of knowledge regarding immunization [22]. As such, vaccine uptake strategies geared toward vaccine-hesitant individuals with adequate health literacy would do well to depart from traditional educational agendas.

### 3.4. Parental Status

Parental status is also an important demographic variable to consider, given that parents do not only make their own vaccination decisions but are also key decision makers for their children with regard to vaccination uptake or refusal. Research shows that people with young children experience more aversion to potential side effects or risks associated with vaccines [18]. Indeed, a Malaysian research team found that the most common reason underlying hesitancy in parents with young children was concern over side effects [20]. Parental status also interacts with other variables to influence vaccination outcomes. Wu et al. found that chronic illness was positively linked with vaccine uptake among parents [27]. In addition to whether one has children, the number of children is also relevant to vaccine-related attitudes. In the U.S., having more than three children in the family is a strong determinant of immunization noncompliance [41]. Likewise, research with Greek parents has found that having 3+ children negatively relates to intentions to vaccinate [31]. These findings, at first blush, seem counterintuitive given that having multiple children should encourage parents to avoid preventable infectious diseases to prevent cross-infection within the household. However, relations between family size and vaccine hesitancy may be explained through interrelated factors, including socioeconomic status [27].

Health care provider recommendations appear to be particularly impactful to parents. Parents whose health provider recommends vaccination are more likely to vaccinate their children [27]. In fact, U.S. parents and their health care providers tend to have very similar beliefs about vaccinations [42]. Parents who are confident in the safety and efficacy of vaccines often have providers who promote full vaccination schedules, whereas vaccine-hesitant parents are more likely to have providers who question the overuse of immunizations. These correlational results may reflect *socialization*, whereby health providers shape parental attitudes or *selection*, where parents ‘shop’ for a provider with similar health beliefs. In either case, these findings highlight the importance of knowledge sharing between health care providers and their patients, particularly for patients who are parents.

Disseminating COVID-19 vaccine information through primary health care providers may be a successful strategy for enhancing public confidence among parents. Parents frequently cite their health care provider as the main source of information on vaccines, although evidence suggests physicians spend minimal time on vaccine-related discussions during appointments [25,27]. Parent-centred information on vaccines could be distributed by primary care providers, using modalities that are efficient and time-effective. These information outlets may take the form of brochures, pamphlets, web-based aids for parents, all of which have been found to positively affect parent intent to vaccinate. Importantly, much of the extant work on parental attitudes has been done with parents of young children, representing a notable knowledge gap given that age guidelines are currently 12+ for the Pfizer/BioNTech vaccine and 18+ for the Moderna vaccine [8,9].

### 3.5. Rurality

Vaccination behaviours are influenced by geographic considerations, including whether individuals live in densely populated areas or rural regions. Longer travel to administration sites has been acknowledged as a barrier to being vaccinated, which may mean reduced uptake among rural populations [31]. Similar results have been found in studies focusing on parental barriers to child vaccination, with mothers living in rural areas of China reporting significantly lower vaccination rates for their children compared to urban households [43]. It is unclear whether different intentions and behaviours regarding vaccination in rural areas reflect inconvenience or lack of confidence. Research with a cohort in the U.K. suggests the latter, finding that individuals living in rural areas report less confidence in vaccinations than those from urban areas [18]. Recent surveys on COVID-19 vaccine hesitancy in the U.S. confirmed that rates among rural residents were higher than the general U.S. public, with 35% of rural respondents reporting that they probably or definitely would not get a COVID-19 vaccine [44]. These discrepancies in rural versus urban settings may be addressed by efforts to boost vaccine confidence targeted to rural regions and by attempts to de-centralize vaccination clinics. Geographical differences in vaccine hesitancy could theoretically lead to disparities in vaccination coverage and localized outbreaks for the foreseeable future.

With the exception of smallpox, it is safe to expect that diseases do not die off. In many countries, widespread immunization programs are not possible for a variety of reasons, some related to geographical limitations, some because of social determinants such as poverty, overhead costs, and lack of understanding. Yet, whenever public health departments have instituted community immunization programs, there has been a concomitant decrease in the incidence rate (number of new cases within a defined period) that can lead to significant declines in illness, hospitalizations, long-term infirmity, and death [38]. Once more, this emphasizes the importance of equitable immunization programs to ensure proportionate uptake across urban and rural regions.

### 3.6. Mistrust in Authority

The level of trust in health and government authorities can shape attitudes toward vaccination in a number of ways. Mistrust is more likely to occur under uncertain conditions when there is a perceived threat among marginalized groups [45]. In this way, mistrust in authority can be seen as a coping mechanism to increase the individual or group’s sense of control and certainty [46]. Indeed, Kennedy (2020) said, ‘Vaccine hesitancy appears to be one aspect of a broader breakdown in trust between some sections of the population on the one hand, and elites and experts on the other’ [29]. Reuben and others found that a general mistrust of the medical profession predicted vaccine hesitancy among parents in Canada, the U.S., and the U.K [47]. Similarly, parents who lack trust in their family physicians are more likely to consult the Internet for advice regarding vaccination, which, in turn, promotes vaccine refusal [48]. U.S. surveys assessing vaccine hesitancy have found that mistrust is a common reason underlying intentions to avoid COVID-19 vaccines, with 55% of hesitant respondents saying they lack trust in the government to ensure vaccine safety and effectiveness [44]. Similarly, survey respondents in the U.K. and Ireland who were COVID-19 vaccine-hesitant were unlikely to obtain information from traditional authorities and reported mistrust in authoritative sources [45].

Mistrust, in its most amplified form, may manifest as conspiracy beliefs. Although conspiracy theories of the COVID-19 pandemic and vaccines may be uncommon, they are extremely visible. Individuals with conspiracy views of the pandemic, in general, and of the vaccine, more specifically, are more likely to voice their opinions than those with more mainstream viewpoints [45]. Self-identified conspiracy beliefs about vaccines have been associated with intentions to refuse vaccines, as have conspiracy beliefs fostered through experimental manipulation, that is, being exposed to information that supported anti-vaccine conspiracy theories [49].

One strategy for targeting vaccine hesitancy that ensues from mistrust in authority is to share knowledge through trusted and relatable sources (i.e., peers). Other areas of public health, such as substance use prevention and intervention, have emphasized peer-led initiatives to communicate information in a way that is palatable and personal. Peer-led outreach activities are highly valued by people who access them, as they increase one’s sense of self-determination and internalize the locus of control [50]. Individuals who are less trusting of authority figures may be more responsive to members of the public sharing their intentions to immunize or sharing information they receive from credible sources. Providing scientific information about the benefits of vaccines has proven ineffective in countering anti-vaccination beliefs [47]. However, perhaps it is not the information but the way it is being transmitted that is ineffective. Incorporating a community voice and highlighting the collaboration between health experts, leaders, and peers could foster trust and transparency in the development and rollout of vaccines.

### 3.7. Disgust Sensitivity

Disgust sensitivity can be conceptualized in a number of ways: as a personality trait, an automatic reaction to disturbing or tainted stimuli, or a personal value. Trait-level disgust sensitivity is characterized by a desire to protect oneself from contamination and disease [51]. It is thought to be closely related to purity values and the desire for the sanctity of the body and mind [52]. Associations between greater disgust sensitivity and vaccine hesitancy have been detected among parents in Canada, the U.S., and the U.K [47]. Other research has shown an association between pathogen disgust sensitivity and vaccine hesitancy among university populations [52]. These findings have been replicated in samples of parents, where strong purity values (another expression of disgust sensitivity) were associated with high levels of vaccine hesitancy [53].

Associations between disgust sensitivity and vaccine hesitancy highlight potential gaps in current public health communications. For instance, disgust-sensitive individuals may respond better to content that uses technical and logical terms and avoids potential triggers for disgust, such as images of needles puncturing skin. Additionally, framing the vaccine-preventable disease, in this case, COVID-19, as more damaging than the vaccine and emphasizing the vaccine’s mechanism for building immunity as a natural bodily response may resonate with individuals with strong purity values. According to Horne et al., vaccine hesitancy was reduced after seeing pictures of children with measles and rubella, cues that could be interpreted as disgust inducing [54]. By using disgust eliciting stimuli to demonstrate the risks of not vaccinating, parents with higher disgust sensitivity may become more motivated to vaccinate. In any case, these findings indicate a need for the inclusion of broader themes in vaccine discussions that extend beyond information provision.

### 3.8. Risk Aversion

Risk aversion is a quality closely linked to trait anxiety, with anxious individuals often making decisions that avoid perceived risk. From an economic perspective, risk aversion can be conceptualized as the tendency to weigh potential losses more heavily than potential gains [55]. However, this phenomenon extends beyond financial decisions to other life domains, including health behaviours. Further, individuals may be more or less sensitive to specific types of risk. For instance, according to the *omission bias*, individuals prefer taking a passive risk (i.e., not vaccinating) to taking a risk through active behaviour [56]. Therefore, individuals who are anxious and thus risk-averse may be inclined to forego vaccinating to avoid unknown side effects rather than vaccinate and avoid preventable disease. Indeed, higher levels of anxiety predicted vaccine hesitancy in Israeli parents [57]. To counter the omission bias, access to vaccines should be made as seamless as possible, and initiatives, such as workplace policies permitting time off for vaccination and the use of mobile clinics to reduce the need for travel should be pursued.

However, fear and anxiety can have the opposite effect by promoting vaccination when individuals perceive the vaccine-preventable disease as being dangerous [58]. In essence, the decision to vaccinate is determined by a weighting of risk in terms of whether potential side effects of vaccination or vaccine-preventable disease are perceived as posing a greater risk to the decision maker [17]. This process of calculating risk is also influenced by region and time, where living in an area or in a time where a specific vaccine-preventable disease is prevalent might shift the preference toward vaccinating [59]. At the same time, anxiety appears to be higher for new vaccines than for more established vaccines, with over 50% of respondents to the WHO SAGE Vaccine Hesitancy Scale responding that they believed ‘new vaccines carry more risks than older vaccines’ [34].

Having concerns over potential COVID-19 vaccine side effects is valid. For instance, according to the National Advisory Committee on Immunization (NACI; 2021), reports of very rare blood clots occurred at a rate of 1 in 100,000 to 1 in 250,000 for viral vector vaccines. Such findings emphasize the need to communicate contraindications and follow guidelines closely for specific vaccines. Documented side effects also speak to the dynamic process of weighing potential risks for various vaccines with risks of not vaccinating. It is noteworthy that fear related to vaccination is not only centred on vaccine side effects but also concerns regarding the risk of COVID-19 exposure at vaccination sites [60].

Evidently, building a sense of safety among the public will be essential to addressing the fears outlined above. Work is needed to ensure that transportation to vaccine clinics and the clinics themselves are organized in a way that limits the opportunity for transmission, which will help address some concerns. Accurate information around the outcomes and risks of the disease versus outcomes and risks of vaccination should be provided to encourage individuals to accurately calculate risk and make decisions accordingly. As such, there remains a need for transparent and accessible data on vaccine efficacy and reactions to help inform decisions. Although the COVID-19 vaccine is a new vaccine and, thus, may garner more anxiety, the devastating and pervasive outcomes of the pandemic may shift public preferences toward vaccinating [34]. Psychological research has demonstrated that the perceived degree of negativity of the COVID-19 situation predicts compliance with public health regulations, which could extend to adherence to vaccine recommendations [61]. Research on risk aversion suggests that vaccine discussions must acknowledge that decision making is not purely cognitive but driven heavily by emotions, especially fear.

### 3.9. Limitations and Future Directions

It should be noted that a number of studies summarized in this review pertain to research on vaccine hesitancy conducted prior to the COVID-19 pandemic, with several exceptions. The reason for this being that vaccine hesitancy in the era of COVID-19 is an emerging area of investigation, with limited peer-reviewed research to date. Given the uniqueness of current circumstances, in terms of the devastating global impact of the virus, the novelty of the pathogen and vaccines, and the age of digital technology and information sharing, it is possible that factors might relate differently with hesitancy for COVID-19 vaccinations than vaccine hesitancy, in general, or vaccine hesitancy for established vaccines (i.e., the influenza vaccine). Research with adapted measures of vaccine hesitancy that assess COVID-19 vaccination hesitancy explicitly is required to confirm or refute this possibility. Indeed, it will be important to determine how individual factors interact with current contextual factors, given that vaccine hesitancy differs across time and vaccines [32]. It was beyond the scope of the review to provide a detailed summary of research related to religion and vaccine hesitancy. However, a notable body of research has examined religiosity as a contributor to vaccine refusal, or vaccine uptake, depending on the particular theological views [62]. Individuals who refuse vaccines due to conflicting religious or moral beliefs are unlikely to adapt their vaccination behaviours as a result of public campaigns [63]. Awareness that religious beliefs may influence vaccination attitudes is important so that health officials and providers can create a respectful, informative, and culturally safe dialogue around vaccinations.

It is important to note that an emergence of variants of the SARS-CoV-2 virus, three of which were described by Burki (2021) as P.1, B.1.351, and B.1.1.7, have become prominent in different countries despite measures to prevent community spread and growing vaccination rates. These variants are known as variants of concern (VOC) and the efficacy of Moderna and Pfizer-BioNTech vaccines have been tested for these VOCs. In each circumstance, the pharmaceutical corporations are reporting their products to be effective, despite a current lack of peer-reviewed literature. However, both companies indicate the need to develop a booster shot, given that all viruses mutate [64]. Research is needed to establish how the development of VOCs influences vaccine uptake and to examine whether vaccination rates will persist or decline with the demands of booster shot regimens. It is possible that the presence of VOCs could become the stimulus for change in ensuring that individuals will complete their vaccine regimen as the willingness to actively engage in efforts to end the pandemic appears to be losing momentum, as noted by reduced rates of vaccination in those communities where public health restrictions have ended.

This review took a broad search strategy, documenting articles that pertained to variations on vaccine hesitancy, such as vaccine intentions, vaccine refusal, and vaccine uptake. As such, the constructs reported are quite broad. However, this approach is consistent with the conceptualization of vaccine hesitancy as reflecting a continuum of diverse attitudes and behaviours [14,65].

## 4. Conclusions

The success of available COVID-19 vaccines will rely on vaccinating a large proportion of the population. Public health experts are hoping for population immunization rates of over 70% to reach herd immunity, with some experts advocating for rates as high as 90% in the adult population. In many communities, there will be a sub-group of individuals that cannot be inoculated because they are too young, have a compromised immune system, or are currently being treated for a specific disease. Therefore, optimal immunization rates for eligible populations exceed the percentages presented above. This target highlights a need for rigorous public health campaigns that speak to individuals who are vaccine-hesitant, not fully opposed but also not fully confident. This review identified demographic and individual differences known to contribute to vaccine hesitancy, with the goal of informing successful COVID-19 vaccination programs. Taken together, this research speaks to a need for an assortment of targeted strategies to reach vaccine-resistant individuals. The rush to roll out the COVID-19 vaccinations must not sacrifice well-planned public health communications and targeted outreach to bolster confidence in those who require it most.

## Figures and Tables

**Table 1:** Summary of key findings organized by predictor and by population.

Predictors of Vaccine Hesitancy	Population	Key Findings
	Older and younger adults in China	Reasons for vaccinating varied by age with susceptibility to disease predicting influenza vaccine uptake in older adults and perceived effectiveness predicting vaccination in younger adults [17].
Age	Adults and young adults in the U.K.	Vaccine attitudes differed across age groups, with adults 50 to 59 reporting more confidence in vaccines than adults 20 to 29 [18]
	Parents in the U.S.; Parents in Malaysia	Younger parental age was associated with vaccine hesitancy as measured by the Parent Attitudes about Childhood Vaccines Questionnaire [19,20]
	Adults in the U.S.; Adults in Germany	Increased social media use contributed to negative vaccine attitudes, and centennials and millennials were the primary users of social media platforms [21–23]
Implications: Observed age group differences in vaccine hesitancy have connotations for developing effective vaccination campaigns. Findings of increased hesitancy among young adults indicate a need for communications targeted at those cohorts (i.e., sharing evidence-based content over social media).		
Socioeconomic Status	Families in the U.S. with children under 6	Families who refused vaccines for their children were more likely to reside in higher-income communities than families who vaccinated [24]
	Parents of dependent children in the U.S.	Parents of under-immunized children were largely middle-class and college-educated [25]
	Families with infants aged 12–23 months in West Africa	Standard of living was associated with the vaccination status of children, with well-off families being more likely to have children who were vaccinated than families living in poverty [26]
	Postpartum mothers in the U.S.	Low income was associated with less trustful attitudes toward vaccination [27]
Implications: Vaccine campaigns should not be reduced to targeting communities based on SES, as vaccine hesitancy is an issue across social classes. Future research is required to explain why income relates differently to vaccination behaviours and attitudes in different samples.		
Education	Canadian parents	Higher educational attainment related to fewer concerns over vaccine safety, according to results of the National Childhood Immunization Coverage Survey [30]
	Greek parents of 6-year-old children	Paternal education of high school or higher predicted age-appropriate immunizations of children [31]
	Families in the U.S. with children under 6	Families who refused vaccines for their children tended to reside in communities with higher educational attainment, based on census data [24]
	Mothers from several low- to middle-income countries	Education was unrelated to vaccine hesitancy in a multi-ethnic sample, using the WHO Vaccine Hesitancy Scale [34]
Implications: Educational campaigns may be effective means for addressing knowledge gaps and correcting misinformation. Public education should explain the mechanisms of action of vaccines using everyday terminology and plain language. Education on the history of transmissible disease and the role of vaccination programs in controlling outbreaks will create pertinent conversations in combatting the COVID-19 pandemic. Vaccine uptake strategies geared toward individuals with adequate education and health literacy should depart from educational agendas and, instead, focus on perceived risks and fears.		
Parental Status	British adults responding to an adapted WHO Vaccine Hesitancy Scale	Participants with young children experienced the most aversion to potential side effects and risks of vaccines [18]
	Parents in Malaysia	The most common reason for vaccine hesitancy among parents with young children was concern over side effects [20]
	Parents in West Africa; Parents in the U.S.; Parents in Greece	Family size was a consistent predictor of vaccine uptake, and families with 3+ children were more likely to refuse immunizations [26,31,41]
	Postpartum mothers in the U.S.; Parents in the U.S.	Parental vaccine decisions were strongly linked to health care provider recommendations. Parents who were vaccine-hesitant were more likely to have care providers who questioned the overuse of immunizations [25,27,42]
Implications: Findings highlight the importance of knowledge sharing between health care providers and parents. Parent-centred information on vaccines should be distributed by primary care providers in ways that are efficient and effective. Brochures, pamphlets, and web-based aids for parents are all evidence-based outlets shown to positively affect parents’ intent to vaccinate. Much of this research has taken place with parents of young children, representing a notable knowledge gap given that age guidelines are currently 12+ for the Pfizer/BioNTech vaccine and 18+ for the Moderna vaccine. ^a^		
Rurality	Parents in Greece	Longer travel time to vaccine administration sites has been cited as a barrier to being vaccinated [31]
	Mothers in rural and urban areas of China	Mothers from rural regions report significantly lower vaccination rates for their children compared to mothers in urban areas [43]
	Adults in the U.K.	Vaccine confidence was stronger among urban residents than individuals residing in rural areas in the U.K [18]
	Adults in the U.S.	Surveys completed in December 2020 revealed greater vaccine hesitancy among rural respondents than the general population, with 35% of rural participants indicating that they would probably not or definitely not get a COVID-19 vaccine [44]
Implications: Discrepancies in rural versus urban settings need to be addressed by efforts to boost vaccine confidence in rural regions and by attempts to decentralize vaccination clinics. Otherwise, geographical differences in vaccine hesitancy could lead to disparities in vaccination coverage and localized COVID-19 outbreaks for the foreseeable future.		
Mistrust in Authority	Parents in Canada, the U.S., the U.K.	Mistrust in the medical profession predicted vaccine hesitancy in parents in Canada, the U.S., and the U.K [47]
	Parents in the U.S.	Individuals who lacked trust in their family physician were more likely to consult the Internet for advice on vaccinations, which, in turn, negatively affected vaccination attitudes and behaviours [48]
	Adults in the U.S.	Mistrust was a common reason for not planning to get a COVID-19 vaccine. Fifty-five percent of survey respondents stated they lacked trust in the government to ensure vaccine safety and effectiveness [44]
	Adults in the U.K.; Adults in Ireland	Adults who self-identified as vaccine-hesitant on a COVID-19 vaccine survey reported mistrust in authorities and a reluctance to obtain information from traditional sources [45]
Implications: One strategy for addressing mistrust is to share knowledge through relatable sources (i.e., peers). Other areas of public health, such as substance use prevention and intervention, have emphasized peer-led initiatives. Individuals experiencing mistrust in government or the health system may be more responsive to members of the public sharing their intentions to immunize or providing information they receive from credible sources. Incorporating a community voice and highlighting collaboration between experts, leaders, and peers could help build vaccine confidence among this population.		
Disgust Sensitivity	Parents in the U.S., U.K., and Canada	Respondents who scored higher on global measures of disgust sensitivity were more vaccine-hesitant, as per scores on the Parent Attitudes about Childhood Vaccines Scale [47]
	Undergraduate university students in the U.S.	Positive associations have been found between pathogen disgust sensitivity and vaccine hesitancy among university students [52]
	Parents in the U.S.	Purity values (another measure of disgust sensitivity) were associated with high levels of vaccine hesitancy among parents. High-hesitancy respondents were over twice as likely to endorse strong purity values [53]
Implications: Associations between disgust sensitivity and vaccine hesitancy highlight a need to integrate broader themes into vaccine discussions. Disgust-sensitive individuals may respond better to content that uses technical and logical terms and avoids potential triggers for disgust, such as images of needles puncturing skin. Emphasizing the vaccine’s mechanism for building immunity as a natural bodily response may resonate with individuals with strong purity values.		
Risk Aversion	Israeli Parents	Trait anxiety is closely linked to risk aversion, and individuals high in anxiety have been found to experience greater levels of vaccine hesitancy [55,57]
	Mothers with young infants	Qualitative research has provided insight into a preference for passive risk (i.e., not vaccinating) over taking a risk through active behaviour, in a phenomenon known as the omission bias [56]
	Adults in low- and middle-income countries	Studies using the WHO Vaccine Hesitancy Scale have found that a majority of people believe new vaccines carry more risk than older vaccines [34]
	Parents and caregivers in Taiwan	Fear and risk aversion can promote vaccination when individuals perceive the vaccine-preventable disease as being prevalent and/or dangerous [58]
Implications: Building a sense of safety among the public will be essential to addressing the outlined fears. Work is needed to ensure that transportation to vaccine clinics and the clinics themselves are organized in a way that limits the opportunity for transmission, which will help address some concerns. Accurate information around the outcomes and risks of the disease versus outcomes and risks of vaccination should be provided to encourage individuals to accurately calculate risk and make informed decisions. Public health communications must acknowledge that decision making is not purely cognitive but driven heavily by emotions, especially fear.		

^a^ In Canada.

## Data Availability

Not applicable.

## References

[1] World Health Organization (WHO) Epidemiological Report 2021. https://www.who.int/publications/m/item/weekly-epidemiological-update---1-december-2020.

[2] Health Canada Covid-19 Outbreak Updates 2021. https://www.canada.ca/en/public-health/services/diseases/coronavirus-disease-covid-19.html.

[3] Walensky R.P., Del Rio C. (2020). From mitigation to containment of the COVID-19 pandemic: Putting the SARS-CoV-2 genie back in the bottle. JAMA.

[4] Wilder-Smith A., Freedman D.O. (2020). Isolation, quarantine, social distancing and community containment: Pivotal role for old-style public health measures in the novel coronavirus (2019-nCoV) outbreak. J. Travel Med..

[5] Health Canada Vaccines and Treatments for Covid-19: Vaccine Rollout 2021. https://www.canada.ca/en/public-health/services/diseases/2019-novel-coronavirus-infection/prevention-risks/covid-19-vaccine-treatment/vaccine-rollout.html.

[6] Jacobson R.M., Sauver J.L.S., Rutten L.J.F. (2015). Vaccine Hesitancy. Mayo Clinic Proceedings.

[7] Heininger U., Bachtiar N.S., Bahri P., Dana A., Dodoo A., Gidudu J., Matos dos Santos E. (2012). The concept of vaccination failure. Vaccine.

[8] Health Canada Moderna Covid-19 Vaccine: What You Should Know 2021. https://www.canada.ca/en/health-canada/services/drugs-health-products/covid19-industry/drugs-vaccines-treatments/vaccines/moderna.html#a3-Canada.ca.

[9] Health Canada Pfizer-BioNTech COVID-19 Vaccine: What You Should Know 2021. https://www.canada.ca/en/health-canada/services/drugs-health-products/covid19-industry/drugs-vaccines-treatments/vaccines/pfizer-biontech.html.

[10] Harrison E.A., Wu J.W. (2020). Vaccine confidence in the time of COVID-19. Eur. J. Epidemiol..

[11] Conrod P.J., O’Leary-Barrett M., Newton N., Topper L., Castellanos-Ryan N., Mackie C., Girard A. (2013). Effectiveness of a selective, personality-targeted prevention program for adolescent alcohol use and misuse: A cluster randomized controlled trial. JAMA Psychiatry.

[12] Hudson A., Thompson K., MacNevin P.D., Ivany M., Teehan M., Stuart H., Stuart H.S. (2018). University students’ perceptions of links between substance use and mental health: A qualitative focus group study. Emerg. Adulthood.

[13] Funk C., Tyson A. (2020). Intent to Get a COVID-19 Vaccine Rises to 60% as Confidence in Research and Development Process Increases. Pew Res. Center.

[14] Greenberg J., Dubé E., Driedger M. (2017). Vaccine hesitancy: In search of the risk communication comfort zone. PLoS Curr..

[15] World Health Organization (WHO) Ten Threats to Public Health in 2019. https://www.who.int/news-room/spotlight/ten-threats-to-global-health-in-2019.

[16] Gates B. (2020). Responding to Covid-19—A once-in-a-century pandemic?. NEJM.

[17] Wu S., Su J., Yang P., Zhang H., Li H., Chu Y., Hua W., Li C., Tang Y., Wang Q. (2017). Factors associated with the uptake of seasonal influenza vaccination in older and younger adults: A large, population-based survey in Beijing, China. BMJ Open.

[18] Luyten J., Bruyneel L., van Hoek A.J. (2019). Assessing vaccine hesitancy in the UK population using a generalized vaccine hesitancy survey instrument. Vaccine.

[19] Opel D.J., Taylor J.A., Mangione-Smith R., Solomon C., Zhao C., Catz S., Martin D. (2011). Validity and reliability of a survey to identify vaccine-hesitant parents. Vaccine.

[20] Azizi F.S.M., Kew Y., Moy F.M. (2017). Vaccine hesitancy among parents in a multi-ethnic country, Malaysia. Vaccine.

[21] Mitra T., Counts S., Pennebaker J. (2016). Understanding anti-vaccination attitudes in social media. Proceedings of the International AAAI Conference on Web and Social Media.

[22] Volkman J.E., Hokeness K.L., Morse C.R., Viens A., Dickie A. (2021). Information source’s influence on vaccine perceptions: An exploration into perceptions of knowledge, risk and safety. J. Commun. Heal..

[23] Fietkiewicz K.J., Lins E., Baran K.S., Stock W.G. Inter-generational comparison of social media use: Investigating the online behavior of different generational cohorts. Proceedings of the 49th Hawaii International Conference on System Sciences.

[24] Wei F., Mullooly J.P., Goodman M., Mccarty M.C., Hanson A.M., Crane B., Nordin J. (2009). Identification and characteristics of vaccine refusers. BMC Pediatrics.

[25] Luthy K.E., Beckstrand R.L., Callister L.C. (2010). Parental hesitation in immunizing children in Utah. Public Health Nurs..

[26] Sia D., Fournier P., Kobiané J.F., Sondo B.K. (2009). Rates of coverage and determinants of complete vaccination of children in rural areas of Burkina Faso (1998–2003). BMC Public Health.

[27] Wu A.C., Wisler-Sher D.J., Griswold K., Colson E., Shapiro E.D., Holmboe E.S., Benin A.L. (2008). Postpartum mothers’ attitudes, knowledge, and trust regarding vaccination. Matern. Child. Health J..

[28] Ambwani S., Sellinger G., Rose K.L., Richmond T.K., Sonneville K.R. (2020). “It’s healthy because it’s natural.” Perceptions of “clean” eating among US adolescents and emerging adults. Nutrients.

[29] Kennedy J. (2020). Vaccine hesitancy: A growing concern. Pediatric Drugs.

[30] Carpiano R.M., Polonijo A.N., Gilbert N., Cantin L., Dubé E. (2019). Socioeconomic status differences in parental immunization attitudes and child immunization in Canada: Findings from the 2013 Childhood National Immunization Coverage Survey (CNICS). Prev. Med..

[31] Danis K., Georgakopoulou T., Stavrou T., Laggas D., Panagiotopoulos T. (2010). Socioeconomic factors play a more important role in childhood vaccination coverage than parental perceptions: A cross-sectional study in Greece. Vaccine.

[32] Larson H.J., De Figueiredo A., Zhao X., Schulz W.S., Verger P., Johnston I., Cook A., Jones N.S. (2016). The state of vaccine confidence 2016: Global insights through a 67-country survey. EBioMedicine.

[33] Vikram K., Vanneman R., Desai S. (2012). Linkages between maternal education and childhood immunization in India. Soc. Sci. Med..

[34] Wagner A.L., Masters N.B., Domek G.J., Mathew J.L., Sun X., Asturias E.J., Ren J., Huang Z., Contreras-Roldan I.L., Gebremeskel B. (2019). Comparisons of vaccine Hesitancy across five low- and middle-income countries. Vaccines.

[35] Montelpare W.J. (2021). Principles of Health- or what the health are you thinking?. Epidemics Pandemics Spread Illn..

[36] Burton D.R. (2002). Antibodies, viruses and vaccines. Nat. Rev. Immunol..

[37] Hoffmann A.R., Proctor L.M., Surette M.G., Suchodolski J.S. (2016). The microbiome: The trillions of microorganisms that maintain health and cause disease in humans and companion animals. Vet. Pathol..

[38] Gangarosa E.J., Galazka A.M., Wolfe C.R., Phillips L.M., FRCPath E.M., Chen R.T., Gangarosa R.E. (1998). Impact of anti-vaccine movements on pertussis control: The untold story. Lancet.

[39] Abdel-Latif M.M. (2020). The enigma of health literacy and COVID-19 pandemic. Public Health.

[40] Olisarova V., Kaas J., Staskova V., Bartlova S., Papp K., Nagorska M., Korucova R., Reifsnider E. (2021). Health literacy and behavioral health factors in adults. Public Health.

[41] Bundt T.S., Hu H.M. (2004). National examination of compliance predictors and the immunization status of children: Precursor to a developmental model for health systems. Mil. Med..

[42] Mergler M.J., Omer S.B., Pan W.K., Navar-Boggan A.M., Orenstein W., Marcuse E.K., Taylor J., Dehart M.P., Carter T.C., Damico A. (2013). Association of vaccine-related attitudes and beliefs between parents and health care providers. Vaccine.

[43] Wagner A.L., Huang Z., Ren J., Laffoon M., Ji M., Pinckney L.C., Sun X., Prosser L.A., Boulton M.L., Zikmund-Fisher B.J. (2020). Vaccine hesitancy and concerns about vaccine safety and effectiveness in Shanghai, China. Am. J. Prev. Med..

[44] Hamel L., Kirzinger A., Munana C., Brodie M. KFF COVID-19 Vaccine Monitor December 2020. www.kff.org/coronavirus-covid-19/report/kff-covid-19-vaccine-monitor-december-2020/.

[45] Freeman D., Waite F., Rosebrock L., Petit A., Causier C., East A., Jenner L., Teale A.L., Carr L., Mulhall S. (2020). Coronavirus conspiracy beliefs, mistrust, and compliance with government guidelines in England. Psychol. Med..

[46] Murphy J., Vallières F., Bentall R.P., Shevlin M., McBride O., Hartman T.K., McKay R., Bennett K., Mason L., Gibson-Miller J. (2021). Psychological characteristics associated with COVID-19 vaccine hesitancy and resistance in Ireland and the United Kingdom. Nat. Commun..

[47] Douglas K.M., Uscinski J.E., Sutton R.M., Cichocka A., Nefes T., Ang C.S., Deravi F. (2019). Understanding conspiracy theories. Political Psychol..

[48] Reuben R., Aitken D., Freedman J.L., Einstein G. (2020). Mistrust of the medical profession and higher disgust sensitivity predict parental vaccine hesitancy. PLoS ONE.

[49] Jones A.M., Omer S.B., Bednarczyk R.A., Halsey N.A., Moulton L.H., Salmon D.A. (2012). Parents’ source of vaccine information and impact on vaccine attitudes, beliefs, and nonmedical exemptions. Adv. Prev. Med..

[50] Jolley D., Douglas K.M. (2014). The effects of anti-vaccine conspiracy theories on vaccination intentions. PLoS ONE.

[51] Greer A.M., Amlani A., Burmeister C., Scott A., Newman C., Lampkin H., Pauly B., Buxton J.A. (2019). Peer engagement barriers and enablers: Insights from people who use drugs in British Columbia, Canada. Can. J. Public Health.

[52] Tybur J.M., Lieberman D., Griskevicius V. (2019). Microbes, mating, and morality: Individual differences in three functional domains of disgust. J. Personal. Soc. Psychol..

[53] Clifford S., Wendell D.G. (2016). How disgust influences health purity attitudes. Political Behav..

[54] Amin A.B., Bednarczyk R.A., Ray C.E., Melchiori K.J., Graham J., Huntsinger J.R., Omer S.B. (2017). Association of moral values with vaccine hesitancy. Nat. Hum. Behav..

[55] Horne Z., Powell D., Hummel J.E., Holyoak K.J. (2015). Countering antivaccination attitudes. Proc. Natl. Acad. Sci. USA.

[56] Charpentier C.J., Aylward J., Roiser J.P., Robinson O.J. (2017). Enhanced risk aversion, but not loss aversion, in unmedicated pathological anxiety. Biol. Psychiatry.

[57] Benin A.L., Wisler-Scher D.J., Colson E., Shapiro E.D., Holmboe E.S. (2006). Qualitative analysis of mothers’ decision-making about vaccines for infants: The importance of trust. Pediatrics.

[58] Noyman-Veksler G., Greenberg D., Grotto I., Shahar G. (2020). Parents’ malevolent personification of mass vaccination solidifies vaccine hesitancy. J. Heal. Psychol..

[59] Chen M.F., Wang R.H., Schneider J.K., Tsai C.T., Jiang D.D.S., Hung M.N., Lin L.J. (2011). Using the health belief model to understand caregiver factors influencing childhood influenza vaccinations. J. Community Health Nurs..

[60] Shapiro G.K., Tatar O., Dube E., Amsel R., Knauper B., Naz A., Perez S., Rosberger Z. (2018). The vaccine hesitancy scale: Psychometric properties and validation. Vaccine.

[61] Bhopal S., Nielsen M. (2020). Vaccine hesitancy in low-and middle-income countries: Potential implications for the COVID-19 response. Arch. Dis. Child..

[62] Zajenkowski M., Jonason P.K., Leniarska M., Kozakiewicz Z. (2020). Who complies with the restrictions to reduce the spread of COVID-19: Personality and perceptions of the COVID-19 situation. Pers. Individ. Differ..

[63] Lane S., MacDonald N.E., Marti M., Dumolard L. (2018). Vaccine hesitancy around the globe: Analysis of three years of WHO/UNICEF Joint Reporting Form data-2015–2017. Vaccine.

[64] Burki T. (2021). Understanding variants of SARS-CoV-2. Lancet.

[65] Navin M.C., Wasserman J.A., Ahmad M., Bies S. (2019). Vaccine education, reasons for refusal, and vaccination behavior. Am. J. Prev. Med..

